# Renoprotection with SGLT2 inhibitors in type 2 diabetes over a spectrum of cardiovascular and renal risk

**DOI:** 10.1186/s12933-020-01163-9

**Published:** 2020-11-22

**Authors:** Francesco Giorgino, Jiten Vora, Peter Fenici, Anna Solini

**Affiliations:** 1 Department of Emergency and Organ Transplantation, Section of Internal Medicine, Endocrinology, Andrology and Metabolic Diseases, University of Bari Aldo Moro, Policlinico, Piazza Giulio Cesare, 11, 70124 Bari, Italy; 2 Diabetes and Endocrinology, University of Liverpool, Liverpool, UK; 3 AstraZeneca, Cambridge, UK; 4 Department of Surgical, Medical, Molecular and Critical Area Pathology, University of Pisa, Pisa, Italy

**Keywords:** Diabetes mellitus, Renal dysfunction, Renal protection, cardiovascular risk

## Abstract

Approximately half of all patients with type 2 diabetes (T2D) develop a certain degree of renal impairment. In many of them, chronic kidney disease (CKD) progresses over time, eventually leading to end-stage kidney disease (ESKD) requiring dialysis and conveying a substantially increased risk of cardiovascular morbidity and mortality. Even with widespread use of renin–angiotensin system blockers and tight glycemic control, a substantial residual risk of nephropathy progression remains. Recent cardiovascular outcomes trials investigating sodium–glucose cotransporter 2 (SGLT2) inhibitors have suggested that these therapies have renoprotective effects distinct from their glucose-lowering action, including the potential to reduce the rates of ESKD and acute kidney injury. Although patients in most cardiovascular outcomes trials had higher prevalence of existing cardiovascular disease compared with those normally seen in clinical practice, the proportion of patients with renal impairment was similar to that observed in a real-world context. Patient cardiovascular risk profiles did not relevantly impact the renoprotective benefits observed in these studies. Benefits were observed in patients across a spectrum of renal risk, but were evident also in those without renal damage, suggesting a role for SGLT2 inhibition in the prevention of CKD in people with T2D. In addition, recent studies such as CREDENCE and DAPA-CKD offer a greater insight into the renoprotective effects of SGLT2 inhibitors in patients with moderate-to-severe CKD. This review outlines the evidence that SGLT2 inhibitors may prevent the development of CKD and prevent and delay the worsening of CKD in people with T2D at different levels of renal risk.

## Introduction

Renal disorders are common in type 2 diabetes (T2D), with approximately 50% of patients developing some degree of renal impairment and an increasing prevalence of both conditions over time [1]. An analysis of the US Diabetes Collaborative Registry revealed that 94% of people with T2D presented with at least one cardiovascular (CV), metabolic, or renal comorbidity, including 20% with chronic kidney disease (CKD) [2].

**Fig. 1:**
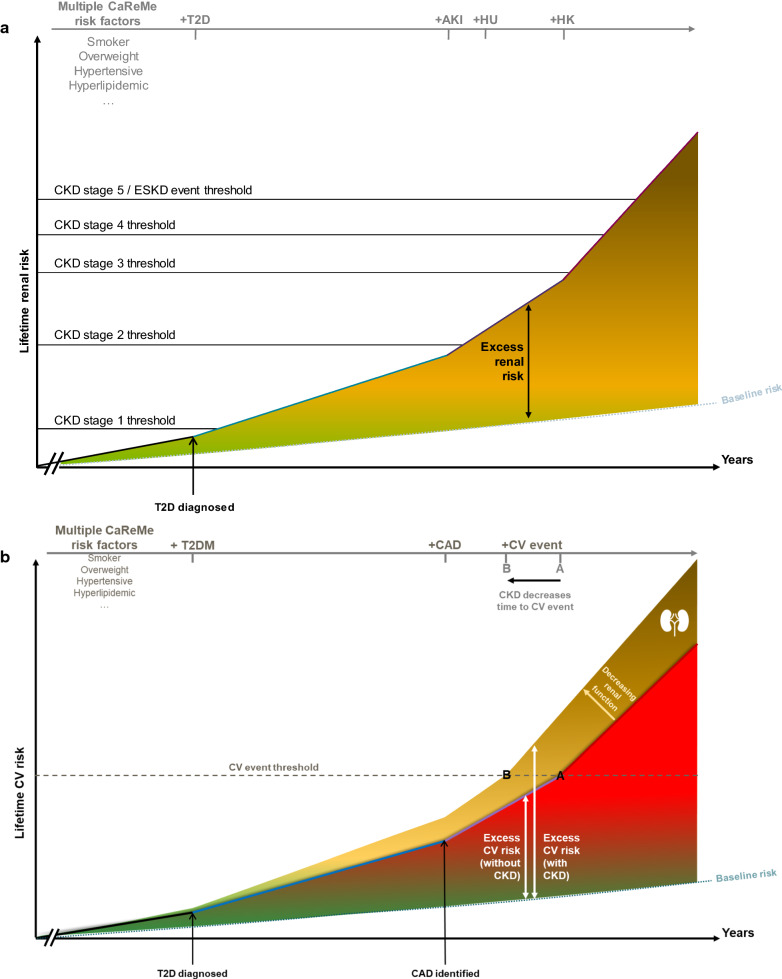
The renal risk spectrum in T2D. **a** In addition to hyperglycemia, a range of other conditions can increase the risk of adverse renal events in T2D, such as AKI incidents, HU, and prolonged HK. **b** CKD amplifies the risk of adverse CV outcomes at any stage. In addition to developing along its own continuum, CKD acts as an amplifier of CV risk, as shown in the rear projection, which highlights that the presence of CKD of any stage increases a person’s CV risk, and that the more advanced the CKD, the greater the increase in CV risk. In effect, this decreases the time taken to cross the CV event threshold (denoted by the leftward shift from **A**, **B**). *AKI* acute kidney injury, *CAD* coronary artery disease, *CaReMe* cardio-renal-metabolic, *CKD* chronic kidney disease, *CV* cardiovascular, *ESKD* end-stage kidney disease, *HK* hyperkalemia, *HU* hyperuricemia, *T2D* type 2 diabetes

The risk of renal disorders in T2D includes the development of multiple phenotypes of organ damage, often overlapping and ultimately progressing, similar to what occurs for cardiovascular disease (CVD) (Fig. 1); many people with T2D already have some degree of renal dysfunction or abnormality at the time of diagnosis, and this may evolve over time, potentially leading to the development of CKD and ultimately to end-stage kidney disease (ESKD) [3, 4]. The UK Prospective Diabetes Study (UKPDS) showed annual accrual rates of approximately 2–3% for the development of microalbuminuria, transition of micro- to macroalbuminuria, and elevated plasma creatinine or need for renal replacement therapy (RRT) [5]. Similarly, in large outcomes trials evaluating angiotensin receptor blockers in people with T2D, progression of nephropathy occurred in approximately 15–27% of placebo-treated patients over 2 years, depending on the level of baseline risk [6–8]. Furthermore, observational studies have identified duration of diabetes as an independent risk factor for progression of renal impairment [9, 10].

Even small decreases in estimated glomerular filtration rate (eGFR) are associated with an increased risk of CV events, including CV mortality [11, 12]. A large-scale analysis (N = 1,120,295) in a cohort not undergoing RRT found that the adjusted hazard ratio (HR) for CV events increased from 1.4 in those with eGFR of 45–59 mL/min/1.73 m^2^ to 3.4 in those with eGFR of < 15 mL/min/1.73 m^2^. Indeed, up to half of deaths in patients with ESKD are thought to be due to CV complications [13]. Therefore, progressive renal impairment increases the risk of patients experiencing not only renal but also CV morbidity and mortality.

Studies of either intensive glucose-lowering therapy [14–18] or specific glucose-lowering drugs versus standard of care [19–24] have consistently shown that correction of hyperglycemia reduces microvascular complications, including nephropathy, in people with T2D. However, the effects of intensive glucose lowering are more evident in terms of reducing albuminuria, as compared with harder renal endpoints such as ESKD, renal death, or fall in eGFR [18]. Moreover, people with T2D may remain at significant risk of progression of CKD despite strict control of both hyperglycemia and hypertension, and the use of therapies such as renin–angiotensin system (RAS) blockers [25].

Sodium–glucose cotransporter 2 (SGLT2) is predominantly expressed in the proximal convoluted renal tubule, and is responsible for approximately 90% of glucose reabsorption by the kidney. Hence, inhibition of this system facilitates the excretion of glucose and sodium in the urine, thereby reducing elevated blood glucose levels, triggering osmotic diuresis, and favoring body weight and blood pressure reductions [25–28]. SGLT2 inhibitors have shown clear CV and renal benefits in people with T2D [22–24, 29–31]. The renoprotective effects of SGLT2 inhibitors across the spectrum of both CV and renal risk are reviewed here.

## Renal benefits of SGLT2 inhibitors in people with T2D

The renoprotective effects of SGLT2 inhibitors in people with T2D have been evaluated in five major CV outcomes trials (CVOTs): EMPA-REG OUTCOME (empagliflozin) [24], CANVAS program, which comprised two randomized, double-blind, placebo-controlled phase 3 trials: CANVAS and CANVAS-R (canagliflozin) [22], DECLARE-TIMI 58 (dapagliflozin) [23], and VERTIS CV (ertugliflozin) [32]. Each of these trials assessed composites of renal events, including ‘hard’ endpoints such as ESKD or renal death, as outcome measures, usually as secondary endpoints. In addition, numerous studies in patients at different levels of renal risk have used measurements such as urinary albumin:creatinine ratio (UACR), eGFR, and serum uric acid levels as overall renal risk markers. Together, these CV outcomes studies indicated that SGLT2 inhibitors could prevent the development of CKD and prevent or delay the worsening of CKD in people with T2D at any level of renal risk. The CREDENCE trial in people with CKD and T2D [33] and DAPA-CKD trial in people with CKD with and without T2D [34] were designed to investigate renal outcomes and demonstrated that SGLT2 inhibitors can reduce the risk of worsening CKD.

### Major outcomes trials with SGLT2 inhibitors in people with T2D at varying levels of CV and renal risk

#### EMPA-REG OUTCOME, CANVAS program, and VERTIS CV

EMPA-REG OUTCOME was a randomized, double-blind, placebo-controlled phase 3 trial, involving 7020 people with T2D, almost all with established CVD [24]. The mean eGFR at baseline was 74.1 mL/min/1.73 m^2^ and the median UACR was 18 mg/g (Table 1). Prespecified renal outcomes in this study included incident or worsening nephropathy (defined as progression to macroalbuminuria, doubling of serum creatinine associated with eGFR < 45 mL/min/1.73 m^2^, initiation of RRT, or death from renal causes) and incident albuminuria [30]. Fewer patients in the empagliflozin group than in the placebo group experienced incident or worsening nephropathy (HR: 0.61; 95% confidence interval [CI]: 0.53, 0.70). A main driver for the reduction in nephropathy with empagliflozin was a slower progression to macroalbuminuria (HR: 0.62; 95% CI 0.54, 0.72). Compared with placebo, empagliflozin was also associated with significant improvements in a *post-hoc* composite endpoint of incident or worsening nephropathy or CV death (HR: 0.61; 95% CI 0.55, 0.69), progression to macroalbuminuria (HR: 0.62; 95% CI 0.54, 0.72), and initiation of RRT (HR: 0.45; 95% CI 0.21, 0.97), with no differences reported between the two therapeutic doses [30].

**Table 1:** Baseline renal status in EMPA-REG OUTCOME, CANVAS program, DECLARE-TIMI 58, VERTIS CV, CREDENCE, and DAPA-CKD

Baseline mean eGFR (mL/min/1.73 m^2^)	EMPA-REG OUTCOME		CANVAS program		DECLARE-TIMI 58		VERTIS CV		CREDENCE		DAPA-CKD^b^	
	74		77		85		76		56		44	
eGFR	N (%)	Mean (SD)^a^	N (%)	Mean (SD)	N (%)	Mean (SD)	N (%)	Mean (SD)	N (%)	Mean (SD)	N (%)	Mean (SD)
≥ 90 mL/min/1.73 m^2^	1538 (21.9)	82.9 (NR)	2476 (24.4)	103.2 (13.2)	8162 (47.6)	98.3 (6.5)	2048 (24.8)	NR	211 (4.8)	NR	348 (12.0)	NR
≥ 60 to < 90 mL/min/1.73 m^2^	3661 (52.2)	48.5 (NR)	5625 (55.5)	74.6 (8.3)	7732 (45.0)	77.0 (8.5)	4390 (53.2)		1558 (35.4)		918 (31.6)	
≥ 45 to < 60 mL/min/1.73 m^2^	1249 (17.8)		1485 (14.6)	53.2 (4.2)	1265 (7.4)	51.4 (7.2)	1807 (21.9)		1266 (28.8)		1239 (42.6)	
≥ 30 to < 45 mL/min/1.73 m^2^	570 (8.1)		554 (5.5)	38.2 (5.1)	–	–	–		1191 (27.1)		401 (13.8)	
≥ 15 to < 30 mL/min/1.73 m^2^	–		–	–	–	–	–		172 (3.9)			
< 15 mL/min/1.73 m^2^	–		–	–	–	–	–		2 (< 0.1)			

Baseline median UACR (mg/g)	18	12	13	NR	927	1017
UACR, N (%)						
Normoalbuminuria (< 30 mg/g)	4171 (60.0)	7007 (69.8)	11,652 (67.9)	NR	31 (0.7)	1 (0.0)
Microalbuminuria (30 to ≤ 300 mg/g)	2013 (29.0)	2266 (22.6)	4023 (23.4)		496 (11.3)	308 (10.6)
Macroalbuminuria (> 300 to ≤ 3000 mg/g)	769 (11.1)	760 (7.6)	1169 (6.8)		3371 (76.6)	2597 (89.4)
Nephrotic range (> 3000 mg/g)	–	–	–		503 (11.4)	1 (0.0)

*eGFR* estimated glomerular filtration rate, *NR* not reported, *SD* standard deviation, *UACR* urine albumin:creatinine ratio [22, 32, 35–38]

^a^For EMPA-REG OUTCOME, mean (SD) eGFRs were only available for patients ≥ 60 mL/min/1.73 m^2^ and < 60 mL/min/1.73 m^2^

^b^For DAPA-CKD, only data from the T2D sub-population is presented

Similar results were observed with canagliflozin in CANVAS and CANVAS-R [22]. These studies included 10,142 people with T2D at various levels of CV risk, a mean baseline eGFR of 76.5 mL/min/1.73 m^2^, and a median UACR of 12 mg/g (Table 1). A secondary renal outcome in CANVAS was progression of albuminuria (≥ 30% increase in albuminuria or change from either normoalbuminuria to microalbuminuria or from microalbuminuria to macroalbuminuria); there was also a prespecified exploratory composite renal outcome (40% decrease in eGFR sustained for ≥ 2 consecutive measurements, need for RRT, or death from renal causes). Progression of albuminuria occurred less frequently with canagliflozin compared with placebo (HR: 0.73; 95% CI 0.67, 0.79), and regression of albuminuria was more frequent (HR: 1.70; 95% CI 1.51, 1.91). The composite renal outcome was also reduced in canagliflozin-treated patients compared with placebo (HR: 0.60; 95% CI 0.47, 0.77) [22].

Unlike EMPA-REG OUTCOME and the CANVAS program, the recent VERTIS CV study investigating ertugliflozin in 8246 people with T2D and established CVD did not show a statistically significant reduction in the secondary composite renal outcome (death from renal causes, RRT, or doubling of serum creatinine levels) in the ertugliflozin group compared with placebo (HR: 0.81; 95% CI 0.63, 1.04) [32]. Mean baseline eGFR of the VERTIS CV population was similar to those of EMPA-REG OUTCOME and the CANVAS program (Table 1); however, owing to differences in outcome definition, renal efficacy outcomes should be compared with caution between trials, and further *post-hoc* analyses of the VERTIS CV data would be useful to provide a more direct comparison [32].

#### DECLARE-TIMI 58

DECLARE-TIMI 58 was a randomized, double-blind, placebo-controlled trial with dapagliflozin, involving people with T2D and either atherosclerotic CVD (n = 6971) or multiple CV risk factors (n = 10,189) [23]. Patients had relatively good renal function, with a mean eGFR at baseline of 85.2 mL/min/1.73 m^2^ and a median UACR of 13 mg/g (Table 1). Compared with placebo, dapagliflozin was associated with significant decreases in both the renal composite endpoint (sustained ≥ 40% decrease in eGFR to < 60 mL/min/1.73 m^2^, new ESKD, or renal or CV death, HR: 0.76; 95% CI 0.67, 0.87) and the additional composite that excluded CV death (HR: 0.53; 95% CI 0.43, 0.66) [23]. Dapagliflozin also decreased the likelihood of new-onset macroalbuminuria (HR: 0.54; 95% CI 0.45, 0.65), new-onset albuminuria (HR: 0.79; 95% CI 0.72, 0.87), and albuminuria category deterioration (HR: 0.73; 95% CI 0.67, 0.79) [39].

#### CREDENCE

**Fig. 2:**
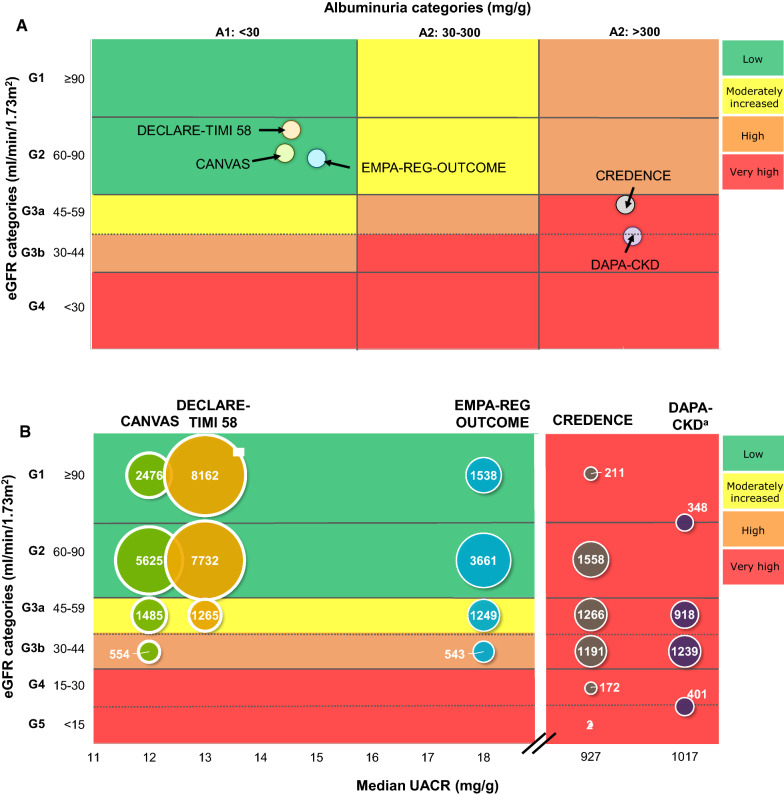
Renal risk in CV and renal outcomes trials with SGLT2 inhibitors. **a** Overall populations; **b** EMPA-REG OUTCOME, CANVAS program, DECLARE-TIMI 58, CREDENCE and DAPA-CKD baseline populations by eGFR group and median UACR. In DECLARE-TIMI 58, people with CrCl of ≤ 60 mL/min were excluded, which led to very few participants with eGFR at baseline < 60 mL/min/1.73 m^2^. ^a^DAPA-CKD T2D subpopulation only. VERTIS CV excluded from this figure because a median UACR was not reported [32]. *CrCl* creatinine clearance, *CV* cardiovascular, *eGFR* estimated glomerular filtration rate, *SGLT2* sodium–glucose cotransporter 2, *UACR* urine albumin to creatinine ratio

The phase 3 CREDENCE trial was the first dedicated renal outcomes trial with an SGLT2 inhibitor, and involved 4401 people with CKD and T2D who were also receiving standard care with RAS inhibitors [33, 40]. The trial recruited people with overt albuminuria (almost 90% had UACR > 300 mg/g to 5000 mg/g), and 59.8% of patients had an eGFR of < 60 mL/min/1.73 m^2^. The baseline renal measures for the trial showed that patients were highly albuminuric (median UACR of 927 mg/g/24 h) with a moderately reduced eGFR (mean eGFR of 56 mL/min/1.73 m^2^) (Table 1, Fig. 2). The relative risk of the primary renal composite endpoint (ESKD, doubling of serum creatinine, or renal or CV death) was 30% lower in the canagliflozin group than in the placebo group (HR: 0.70; 95% CI 0.59, 0.82) [33, 41]. In canagliflozin-treated patients, the relative risks of the composite endpoint without CV death (HR: 0.66; 95% CI 0.53, 0.81) and ESKD (HR: 0.68; 95% CI 0.54, 0.86) were also lower compared with placebo [33].

#### DAPA-CKD

DAPA-CKD was the first phase 3 trial to investigate the safety and renal outcomes of an SGLT2 inhibitor in people with CKD, both with and without T2D [34]. It recruited 4304 people with CKD, of whom 2906 had comorbid T2D. All patients received standard care with RAS inhibitors and had reduced renal function, with a mean baseline eGFR of 43.1 mL/min/1.73 m^2^ (43.8 mL/min/1.73 m^2^ in the T2D subpopulation); 48.3% of patients had a UACR of > 1000 mg/g (median UACR in the T2D subpopulation was 1016.2 mg/g, Table 1). Similar significant reductions in the primary renal composite endpoint (eGFR < 50%, ESKD, or renal or CV death) were observed for patients treated with dapagliflozin compared with placebo, both in those with T2D (HR: 0.64; 95% CI 0.52, 0.79) and those without T2D (HR: 0.50; 95% CI 0.35, 0.72). A significant reduction in the relative risk of the composite endpoint without CV death (HR: 0.56; 95% CI 0.45, 0.68) and all-cause mortality (HR: 0.69; 95% CI 0.53, 0.88) was also reported in the dapagliflozin arm compared with the placebo arm [34, 38].

#### Meta-analyses of trials with SGLT2 inhibitors

Two independent meta-analyses of the EMPA-REG OUTCOME, CANVAS program, DECLARE-TIMI 58, and CREDENCE trials found that SGLT2 inhibitors significantly reduced the risk of a renal composite including worsening renal function, ESKD, or death from renal causes compared with placebo (relative risk [RR]: 0.67; 95% CI 0.52, 0.86 [42] and RR: 0.63; 95% CI 0.56, 0.71; number needed to treat [NNT] = 67) [43]. These outcomes were consistent with results from an earlier meta-analysis, which did not include CREDENCE [44].

These findings were also consistent with those of a meta-analysis of 27 studies involving 7363 patients treated with SGLT2 inhibitors (empagliflozin, dapagliflozin, canagliflozin, and ertugliflozin) [45], which revealed a significantly reduced risk of doubling of serum creatinine (or 40% decline in eGFR, in the case of DECLARE-TIMI 58), ESKD, or renal death (HR: 0.71; 95% CI 0.53, 0.95). In addition, SGLT2 inhibitors significantly reduced the annual decline in eGFR slope, with a mean treatment difference of 1.35 mL/min/1.73 m^2^/year [45]. An additional meta-analysis of 51 randomized controlled trials including a total of 24,163 people with T2D observed no significant differences between treatment with high- and low-dose SGLT2 inhibitors in terms of renal-related adverse events, although composite renal outcomes were not investigated [46].

## Renoprotective effects of SGLT2 inhibitors according to baseline risk

### Renal benefits according to baseline CV risk

One meta-analysis of EMPA-REG OUTCOME, CANVAS program, and DECLARE-TIMI 58 showed that the renal benefit was observed both in patients with (HR: 0.56; 95% CI 0.47, 0.67) and without (HR: 0.54; 95% CI 0.42, 0.71) established atherosclerotic CVD, with no significant difference in effect between the two cohorts (*P* = 0.71 for interaction) [44].

Similarly, *post-hoc* analyses from the CANVAS program and DECLARE-TIMI 58 reported similar decreases in composite renal risk in patients with (canagliflozin: HR: 0.67; 95% CI 0.3, 1.51; dapagliflozin: HR: 0.58; 95% CI 0.36, 0.92) and without (canagliflozin: HR: 0.52; 95% CI 0.31, 0.72; dapagliflozin: HR: 0.52; 95% CI 0.41, 0.66) heart failure at baseline (*P* = 0.93 and *P* = 0.78 for interaction, respectively) [36, 47]. Studies investigating SGLT2 inhibitors in patients with heart failure with reduced ejection fraction (with and without T2D) have also recently reported numerical and substantial reductions in renal composite outcomes compared with placebo [dapagliflozin: HR: 0.71; 95% CI 0.44, 1.16 (eGFR < 50%, ESKD, or renal death) [48]; empagliflozin: HR: 0.50; 95% CI 0.32, 0.77 (eGFR < 50% or ESKD)] [49]. A subanalysis of the DECLARE-TIMI 58 trial found a slightly greater reduction in the rate of a composite of 40% decline in eGFR, ESKD, or renal or CV death in patients without (HR: 0.75; 95% CI 0.64, 0.89) compared with those with (HR: 0.80; 95% CI 0.63, 1.01) prior myocardial infarction, however, the difference was not statistically significant (*P* = 0.69 for interaction) [50].

Subgroup analyses from EMPA-REG OUTCOME and DECLARE-TIMI 58 reported similar renal outcomes irrespective of body mass index (BMI) category with empagliflozin and dapagliflozin therapy for people with T2D [30, 36]. The same observation was reported for the CREDENCE study of canagliflozin therapy for patients with diabetic nephropathy (BMI < 30 kg/m^2^: HR: 0.71; 95% CI 0.56, 0.80; BMI ≥ 30 kg/m^2^: HR: 0.68; 95% CI 0.54, 0.86) [33].

### Renal benefits according to baseline renal risk

Patients in the major outcomes trials differed in terms of their baseline renal risk as measured by eGFR and UACR (Table 1, Fig. 2). When compared, event rates for the renal composite endpoint were highest in the placebo arm of CREDENCE (40.4 per 1000 patient-years) and there were slightly more events in the placebo arms of VERTIS CV (12 per 1000 patient-years), the CANVAS program (9.0 per 1000 patient-years), and EMPA-REG OUTCOME (11.5 per 1000 patient-years) compared with DECLARE-TIMI 58 (7.0 per 1000 patient-years; Table 2).

**Table 2:** Renal composite event rates in EMPA-REG OUTCOME [35], CANVAS program [51], DECLARE-TIMI 58 [36], VERTIS CV [32], CREDENCE [33], and DAPA-CKD [34]

	Treatment arm	Cardiovascular outcomes trials					Renal outcomes trial	
		EMPA-REG OUTCOME	CANVAS program	DECLARE-TIMI 58	Meta-analysis (fixed-effects model)^a^	VERTIS CV	CREDENCE	DAPA-CKD^b^
Renal composite (Event rate per 1000 patient-years)	Placebo	11.5	9.0	7.0	Events (n/N): 766/34,322	12	40.4	NR
	SGLT2i	6.3	5.5	3.7		9	27.0	NR
Hazard ratio (95% CI)		0.54 (0.40, 0.75)	0.60 (0.47, 0.77)	0.53 (0.43, 0.66)	0.55 (0.48, 0.64)	0.81 (0.63, 1.04)	0.66 0.53, 0.81)	0.64 (0.52, 0.79)
Renal composite (3-year NNT)		66	97	103	–	–	28	NR

*CI* confidence interval, *NNT* number needed to treat, *SGLT2i* sodium–glucose cotransporter 2 inhibitor

^a^Meta-analysis does not include VERTIS CV [44]

^b^Outcomes for the T2D subgroup analysis. Renal composite was defined as worsening of renal function, end-stage renal disease, or renal death

Glycemic control resulting from SGLT2 inhibition relies on normal renal function, and hence decreased renoprotection might be anticipated in patients with renal impairment. However, there is evidence that SGLT2 inhibitors actually retain their renoprotection in people with T2D with impaired kidney function. *Post-hoc* analysis of the EMPA-REG OUTCOME trial reported no significant heterogeneity in the treatment effect of empagliflozin on a renal composite endpoint (serum creatinine doubling, RRT, or renal death) among patients according to baseline eGFR (*P* for interaction = 0.51) [30]. However, compared with placebo, empagliflozin reduced the rate of acute renal failure and acute kidney injury (AKI) by 3.1% and 1.5%, respectively, in patients with baseline eGFR < 60 mL/min/1.73 m^2^ and 0.7% and 0.4%, respectively, in those with normal renal function [30]. Although no significant interaction according to baseline UACR was identified for the impact of empagliflozin on the renal composite endpoint (*P* for interaction = 0.18) [30], a *post-hoc* analysis reported that, compared with placebo, empagliflozin therapy attenuated eGFR decline to a greater extent in patients who had macroalbuminuria at baseline than in those with normoalbuminuria or microalbuminuria [52].

A *post-hoc* subgroup analysis of the CANVAS program reported that the impact of canagliflozin versus placebo on the composite renal outcome was larger in patients with macroalbuminuria (HR: 0.48; 95% CI 0.31, 0.74) and normoalbuminuria (HR: 0.50; 95% CI 0.33, 0.77) compared with those with microalbuminuria (HR: 0.98; 95% CI 0.60, 1.60; *P* for heterogeneity = 0.03) [53]. Similarly, although canagliflozin attenuated eGFR decline compared with placebo across all baseline levels of albuminuria, the treatment impact was greatest in patients with macroalbuminuria at baseline [53]. The authors suggested that canagliflozin could be particularly efficacious in patients with kidney disease driven by hyperfiltration or changes in vascular function, both of which lead to macroalbuminuria, although the lack of efficacy in patients with microalbuminuria was suggested to be a chance finding [53]. No significant interactions were confirmed, however, treatment with canagliflozin also led to the greatest numerical decrease in the rate of the renal-specific composite outcomes versus placebo in CREDENCE patients with baseline eGFR 45 to < 60 mL/min/1.73 m^2^ (HR: 0.47; 95% CI 0.31, 0.72) compared with those with a baseline eGFR 30 to < 45 mL/min/1.73 m^2^ (HR: 0.71; 95% CI 0.53, 0.94) and 60 to < 90 mL/min/1.73 m^2^ (HR: 0.81; 95% CI 0.52, 1.26; *P* for interaction = 0.18), and in those with baseline UACR > 1000 mg/g (HR: 0.61; 95% CI 0.49, 0.76) compared with those with UACR ≤ 1000 mg/g (HR: 0.90; 95% CI 0.54, 1.50, *P* for interaction = 0.16) [33, 54]. The largest absolute risk reductions were also seen in patients with the lowest baseline eGFR subgroups (*P* heterogeneity = 0.03) [54].

In a population with relatively less renal impairment, DECLARE-TIMI 58 did not report a significant difference in the efficacy of dapagliflozin as measured by the renal composite outcome based on patient baseline eGFR or UACR, although there were some numeric differences suggesting that the renoprotective effects of dapagliflozin may be slightly larger in patients with normal renal function (eGFR ≥ 90 mL/min/1.73 m^2^: HR: 0.50; 95% CI 0.34, 0.73; and eGFR 60 to < 90 mL/min/1.73 m^2^: HR: 0.54; 95% CI 0.40, 0.73; eGFR < 60 mL/min/1.73 m^2^: HR: 0.60; 95% CI 0.35, 1.02) and those with macroalbuminuria (UACR > 300 mg/g: HR: 0.38; 95% CI 0.25, 0.58; UACR < 30 mg/g: HR: 0.52; 95% CI 0.37, 0.74; and UACR 30–300 mg/g: HR: 0.59; 95% CI 0.39, 0.87) [36]. Approximately 13.1% of DECLARE-TIMI 58 participants had CKD (defined as eGFR < 60 mL/min/1.73 m^2^, UACR > 300 mg/g, or both) at baseline [36]. In these patients, dapagliflozin treatment was associated with a significantly lower rate of ESKD or renal death, compared with placebo (HR: 0.41; 95% CI 0.20, 0.82), which was mostly driven by a lower rate of ESKD (HR: 0.31; 95% CI 0.13, 0.79) [36].

In a meta-analysis of these trials, the renoprotective effects of SGLT2 inhibitors were consistent across all levels of baseline eGFR, although the greatest benefits were observed in patients with preserved renal function at baseline: the incidence of the renal composite was reduced by 63% in patients with a baseline eGFR ≥ 90 mL/min/1.73 m^2^, by 40% in those with eGFR 60–90 mL/min/1.73 m^2^, by 45% in those with eGFR 45–60 mL/min/1.73 m^2^, and by 30% in those with eGFR < 45 mL/min/1.73 m^2^ [42]. However, it should be noted that the majority of patients with high eGFR were recruited into the DECLARE-TIMI 58 trial and the majority of patients with low eGFR were recruited into CREDENCE (Table 1, Fig. 2), therefore the possibility of variation between different agents and/or study design cannot be dismissed. Treatment benefit was consistent irrespective of baseline albuminuria (*P* trend = 0.66) and of RAS blockade (*P* heterogeneity = 0.31) [42]. No specific subgroup analysis has been reported to date, however, the DAPA-CKD trial of dapagliflozin included patients with eGFR 25 to < 30 mL/min/1.73 m^2^, comprising 14.5% of the total patient population (both with and without T2D) [34]. The EMPEROR-reduced trial of empagliflozin for the treatment of heart failure in people with and without T2D also did not exclude patients with eGFR 20 to < 30 mL/min/1.73 m^2^ [49]. The initial results from these studies suggest that the renoprotective effects of SGLT2 inhibitors can still be observed in patients with severe CKD.

### Other studies showing effects of SGLT2 inhibitors on specific renal outcomes

#### Effects on eGFR

A number of studies have reported significant stabilization of eGFR following treatment with SGLT2 inhibitors, particularly in comparison with sulfonylureas glimepiride and glipizide [55–58]. A meta-analysis of 40 randomized trials, involving almost 30,000 patients, found that SGLT2 inhibition in patients with renal impairment was associated with an initial decline in eGFR, compared with placebo, which was followed by a return to baseline levels, whereas there was no significant change in eGFR in patients without renal impairment [59]. This meta-analysis was published prior to DECLARE TIMI-58 results, which demonstrated an initial decrease in eGFR compared to placebo in patients with and without renal impairment [36]. Some studies have reported slightly increased rates of renal impairment and renal failure events during SGLT2 inhibitor therapy. In the short term, greater declines in eGFR have been reported with dapagliflozin compared with placebo, after 12 weeks of treatment (− 10.8% versus − 2.9%, respectively) [60]. A further study reported a higher rate of renal failure events with dapagliflozin, compared with glipizide (5.9% versus 3.4%, respectively), the majority of which were manifested as reduced calculated renal creatinine clearance [58]. A study involving 808 low-risk people with T2D showed similar rates of renal impairment and renal failure in the placebo and dapagliflozin groups, although there was a tendency towards increasing rates with higher doses of dapagliflozin [61]. Another study reported higher rates of renal impairment and renal failure events with dapagliflozin 2.5 mg and 5 mg (4.4% and 2.9%, respectively), compared with placebo (1.5%), although the incidence with dapagliflozin 10 mg was equal to that with placebo [62]. A 24-week Japanese study of ipragliflozin in people with T2D reported larger changes in eGFR from baseline for ipragliflozin versus placebo in patients with moderate renal impairment (eGFR 30 to < 60 mL/min/1.73 m^2^) compared with those with mild impairment (eGFR 60 to < 90 mL/min/1.73 m^2^; − 2 mL/min/1.73 m^2^ vs − 0.5 mL/min/1.73 m^2^) [63]. Other studies have reported no significant changes in eGFR during longer term treatment with empagliflozin [64–66], canagliflozin [67–69], dapagliflozin [60, 70, 71], ipragliflozin [72, 73], ertugliflozin [74], or tofogliflozin [75]. All SGLT2 inhibitor CVOTs report an initial rapid reduction in eGFR, followed by a slower decline, or stabilization, in the SGLT2 inhibitor group compared with placebo, explaining the greater decline in eGFR after 12 weeks that was reported in some studies. A meta-analysis including 48 studies of 34,661 patients treated with SGLT2 inhibitors (canagliflozin, dapagliflozin, empagliflozin, ipragliflozin, tofogliflozin) found substantial variation for the treatment impact on change in eGFR across included studies with duration < 52 weeks in the short term, but demonstrated that SGLT2 inhibitors significantly slowed the decline in eGFR in patients with treatment duration > 52 weeks [76].

#### Effects on UACR

In the EMPA-REG H2H-SU trial, the use of empagliflozin as an add-on to metformin was associated with a slight reduction in albuminuria progression, as measured by UACR, compared with glimepiride [55]. Regression of albuminuria in patients receiving empagliflozin, compared with glimepiride, as add-on therapy to metformin, was more pronounced in patients with higher albuminuria at baseline than in those with less impairment. The mean [standard deviation (SD)] changes in UACR from baseline in patients with normoalbuminuria were − 6.7 (37.7) mg/g and 8.6 (72.6) mg/g for the empagliflozin and glimepiride groups, respectively; corresponding values were − 9.0 (98.4) mg/g and 56.5 (677.0) mg/g, respectively, for those with microalbuminuria, and − 483.5 (613.7) mg/g and 380.1 (1161.5) mg/g, respectively, for those with macroalbuminuria [55].

In a 52‐week, multicenter, randomized trial including 269 people with T2D and CKD stage 3, canagliflozin 100 mg and 300 mg resulted in larger decreases in UACR, compared with placebo, at week 26 (− 29.9%, − 20.9%, and − 7.5%, respectively) [77]. Albuminuria progression rates from baseline to week 26 were significantly lower in canagliflozin-treated patients, compared with placebo, with odds ratios (ORs) of 0.33 (95% CI 0.08, 1.48) for the 100 mg dose and 0.51 (95% CI 0.14, 1.91) for the 300 mg dose [78]. At week 52, albuminuria regression rates in patients receiving canagliflozin 100 mg, canagliflozin 300 mg, or placebo were 15.4%, 20.6%, and 11.4%, respectively; these values were slightly higher than the corresponding albuminuria progression rates (10.3%, 14.7%, and 17.1%, respectively) [78]. In CANTATA-SU, UACR decreased with canagliflozin at both 52 weeks [mean (SD)] changes from baseline with canagliflozin 100 mg and 300 mg: − 0.1 [4.7] g/mol and − 0.9 [6.7] g/mol, respectively) and 104 weeks (− 0.02 g/mol, − 0.27 g/mol, respectively), but increased in glimepiride-treated patients (mean increase 0.7 [SD: 15.3] g/mol at week 52, and 1.55 g/mol at week 104) [56].

Mean reductions in UACR in people with T2D receiving dapagliflozin or glipizide as add-on therapy to metformin have been reported as − 19.0 [standard error (SE): 6.6] mg/g and − 0.8 (SE: 7.1) mg/g, respectively [58]. A Japanese study of 86 people with T2D also reported a substantial decrease in log-UACR of 0.37 ± 0.73 over 24 weeks, which was significantly associated with improvements in blood pressure [79]. In a separate *post-hoc* analysis of 166 people with T2D with CKD stage 3 and albuminuria (≥ 30 mg/g), dapagliflozin treatment was associated with a change in UACR at week 104 of − 43.9% (95% CI − 64.3, − 12.0) and − 26.4% (− 55.0, 20.5) at doses of 10 mg and 5 mg, respectively, whereas UACR increased by 31.0% (− 19.0, 111.9) in the placebo group [80]. Another *post-hoc* analysis, using pooled data from two phase 3 trials, assessed people with T2D with inadequately controlled hypertension despite RAS inhibitor therapy. Dapagliflozin significantly reduced albuminuria by 33.2% (95% CI − 45.4, − 18.2%), compared with placebo, and the effect was similar in patients with baseline microalbuminuria (− 35.4%; 95% CI − 48.8, − 18.7%) or macroalbuminuria (− 28.3%; 95% CI − 52.9, 9.2%) [81].

In the DERIVE study, which investigated the effects of dapagliflozin in people with T2D and CKD stage 3a, dapagliflozin did not reduce UACR from baseline at week 24 in the overall population (difference versus placebo: 8.0%; 95% CI − 14.4, 36.3). However, in patients with baseline UACR ≥ 30 mg/g, dapagliflozin significantly reduced UACR from baseline at week 4 (mean treatment difference: − 30.7%; 95% CI − 47.3, − 8.9) and week 12 (mean treatment difference: − 41.7%; 95% CI − 57.1, − 21.0), suggesting added benefit for patients with higher renal risk [82]. The DELIGHT study of people with T2D and UACR ≥ 30 mg/g also reported a decrease in UACR, compared with placebo, at week 24 in patients treated with dapagliflozin (− 21.0%; 95% CI − 34.1, − 5.2) and a combination of dapagliflozin and saxagliptin (− 38.0%, 95% CI − 48.2, − 25.8) [83]. In a pooled analysis of 11 phase 3 clinical trials in patients with no renal impairment or CKD stage 1–3a, dapagliflozin was associated with placebo-corrected reductions in UACR irrespective of baseline eGFR; however, in patients with UACR ≥ 30 mg/g at baseline, the greatest reductions were seen in the subgroup of patients with the lowest eGFR (≥ 45 to < 60 mL/min/1.73 m^2^) [84]. Similarly, in a pooled analysis of 11 randomized trials in patients with CKD stage 3b–4, dapagliflozin 5 mg and 10 mg led to changes in UACR of − 47.1% (95% CI − 64.8, − 20.6) and − 38.4% (95% CI − 57.6, − 10.3), respectively, compared with placebo [85]. Similar results were seen in the subgroup of patients with baseline UACR ≥ 30 mg/g and in subgroups stratified by median albumin concentrations at baseline. Various meta-analyses found that irrespective of the presence or absence of renal impairment, SGLT2 inhibitor therapy was associated with reductions in urine albumin and progression to macroalbuminuria [42, 59, 76].

In contrast to these positive results, some studies have found no significant benefit, in terms of markers of renal function, with SGLT2 inhibitors in people with T2D at low renal risk. An analysis of 12 randomized, double-blind clinical trials involving 4545 patients found that more than 90% of patients, whose baseline renal function was largely preserved or only mildly compromised (eGFR 84.6–86.7 mL/min/1.73 m^2^), remained in the normal albuminuria category in both dapagliflozin and placebo groups, with the proportion of patients with worsening albuminuria at week 24 similar for dapagliflozin and placebo irrespective of baseline eGFR category (normal renal function and mild or moderate renal impairment) [29]. A dose-dependent increase in microalbuminuria has also been reported in a single phase 2 study with empagliflozin in people with T2D [86]. Over 24 weeks, treatment with ipragliflozin was also found to reduce UACR to a lesser degree in patients with mild renal impairment (− 1.28 mg/g versus placebo) compared with those with moderate impairment (− 55.18 mg/g versus placebo) [63]. In addition, dapagliflozin therapy was associated with a non-significant reduction in UACR compared to placebo [mean difference: − 17.0% (95% CI − 33.2, 3.4)] in people with CKD without T2D [87].

### Effects of SGLT2 inhibitors on uric acid

A systematic review found the following reductions in serum uric acid levels with SGLT2 inhibitors compared with placebo or standard care: canagliflozin weighed mean difference (WMD): − 36.72 µmol/L (95% CI − 38.12, − 35.33), dapagliflozin WMD: − 38.05 µmol/L (95% CI − 44.47, − 31.62), and empagliflozin WMD: − 42.07 µmol/L (95% CI − 46.27, − 37.86) [88] This is an important finding because studies with allopurinol and other uric acid-lowering agents have suggested that decreasing uric acid is itself renoprotective [89–93], with a number of potential mechanisms proposed, including the prevention of formation and subsequent adherence of uric acid crystals onto renal epithelial cells and the resulting inflammatory response [94], and the reduction of glomerular hypertension by preventing uric acid-induced vascular smooth muscle proliferation in the afferent arterioles [95, 96]. In EMPA-REG H2H-SU, empagliflozin reduced uric acid levels by 52 (SD: 82) μmol/L from baseline, whereas glimepiride-treated patients showed a mean increase of 16 (SD: 90) μmol/L [55]. Likewise, in a study involving 814 people with T2D and low CV and renal risk, the use of dapagliflozin as add-on therapy to metformin was associated with a mean reduction of uric acid from baseline of 45.2 (SE: 3.4) μmol/L, compared with an increase of 16.1 (SE: 3.4) μmol/L in glipizide-treated patients [58]. Similar results were obtained in a 102-week randomized study with dapagliflozin in low-risk metformin-treated people with T2D [97]. Reductions in uric acid suggestive of improvements in kidney function have also been reported in a number of other studies with empagliflozin [64–66], canagliflozin [67–69, 98], and ipragliflozin [99]. However, other trials have reported no notable differences in various measures of renal function, including serum creatinine and uric acid, with canagliflozin [69, 98], dapagliflozin [70, 71], or tofogliflozin [75, 100].

One reason for these conflicting results might be that trials investigating low-risk patients may be unlikely to reach statistical significance, particularly in subgroup analyses and for secondary or exploratory endpoints, owing to low numbers of observed events and hence low statistical power for tested outcomes. As a result, these findings should be interpreted with caution.

### Potential renal benefits of SGLT2 inhibitors in the real-world setting

While the results from clinical trials, including the CVOTs, provide important insights into the potential benefits of SGLT2 inhibitor therapy, restricted inclusion criteria may limit their generalizability to real-world people with T2D, especially those at the low end of the CV and renal risk spectrum. A European observational study found that the DECLARE-TIMI 58 study most closely resembles the common T2D population particularly in terms of CV risk profile, with 59% representativeness, compared with 34% for the CANVAS program and 21% for EMPA-REG OUTCOME [101]. However, the proportion of people with T2D with low eGFR (< 60 mL/min/1.73 m^2^) was 7.4% in DECLARE-TIMI 58 [23], 26% in EMPA-REG OUTCOME [24], 16.4% in CANVAS [102], and 22.8% in CANVAS-R [103], compared with 18.5% seen in UK general practice [80].

Real-world observational studies can provide important insights into the effectiveness of SGLT2 inhibitors in preventing mortality and morbidity in people with T2D across the entire renal risk spectrum. In general, the results of such studies support those of the major outcomes trials, even though in most countries, SGLT2 inhibitors are not prescribed in patients with eGFR < 60 mL/min/1.73 m^2^, limiting the possibility to extend these observations to a T2D population at higher renal risk. In people with T2D and hypertension, changes in albuminuria occurred in parallel with changes in adverse renal outcomes in an observational study [104], and other studies have shown that UACR is a strong predictor of albuminuria progression in people with T2D [105]. Baseline prevalence of microalbuminuria and macroalbuminuria in EMPA-REG OUTCOME (28.7% and 11%, respectively) [30] and the CANVAS program (23% and 8%, respectively) [106] were also similar to that reported in real-world studies [105, 107].

CVD-REAL 3, a large-scale multinational, observational cohort study including data from 65,231 patients, compared outcomes following initiation of SGLT2 inhibitors (dapagliflozin, empagliflozin, canagliflozin, ipragliflozin, tofogliflozin, and luseogliflozin) with those following initiation of other glucose-lowering drugs (dipeptidyl peptidase-4 [DPP-4] inhibitors, insulin, glucagon-like peptide-1 receptor agonists [GLP-1 RAs], sulfonylurea, thiazolidinedione, metformin, metiglinides, and acarbose) in propensity-matched patient cohorts [108]. Consistent with what was observed in clinical trials, during the mean follow-up of 14.9 months, initiation of SGLT2 inhibitors was associated with a 51% reduction in the risk of 50% eGFR decline or ESKD events, compared with other glucose-lowering drugs (HR: 0.49; 95% CI 0.35, 0.67) [108]. There were no statistically significant differences in the relative risk reductions according to patient baseline eGFR or albuminuria status. SGLT2 inhibitor initiation was also associated with lower risk of hospitalization for heart failure (HR: 0.60; 95% CI 0.47, 0.76) and all-cause mortality (HR: 0.55; 95% CI 0.48, 0.64) [108]. Another recent real-world study, involving approximately 12,000 people with T2D, also investigated renal events following initiation of SGLT2 inhibitors (n = 6418) or DPP-4 inhibitors (n = 5604) [109]. Similar to the results of CVD-REAL 3, compared with DPP-4 inhibitors, SGLT2 inhibitor therapy was associated with decreased risks of ≥ 30% reduction in eGFR (OR: 0.70; 95% CI 0.49, 1.00), AKI (OR: 0.47; 95% CI 0.27, 0.80), hospitalization (OR: 0.66; 95% CI 0.56, 0.78), and all-cause mortality (OR: 0.43; 95% CI 0.20, 0.95) [109].

A real-world Japanese study has investigated the renoprotective effects of SGLT2 inhibitors in 42 people with T2D and moderate-to-severe CKD (stage 3b–4) [110]. After 1 year, the annual decline in eGFR was significantly reduced by SGLT2 inhibitor therapy, as demonstrated by a median change in eGFR from − 3.8 mL/min/1.73 m^2^/year to 0.1 mL/min/1.73 m^2^/year [110]. CVD-REAL 3 also reported a that the rate of eGFR decline was 1.53 mL/min/1.73 m^2^/year less in patients receiving SGLT2 inhibitors compared with those treated with other glucose-lowering therapies [108]. The Japanese study also found that SGLT2 inhibitor therapy was associated with a significant decrease in median UACR, from 0.36 to 0.23 g/g _creatinine_ [110]. Similarly, an Italian real-world study reported that, when adjusted for baseline differences between the groups, 6 months of treatment with dapagliflozin resulted in a reduction in albumin excretion rate, which was 26.4 mg/g greater than that experienced by patients receiving other glucose-lowering medications (combined GLP-1 RAs, DPP-4 inhibitors, or gliclazide; *P* = 0.049) [111].

### Mechanisms of renal benefits with SGLT2 inhibitors

**Fig. 3:**
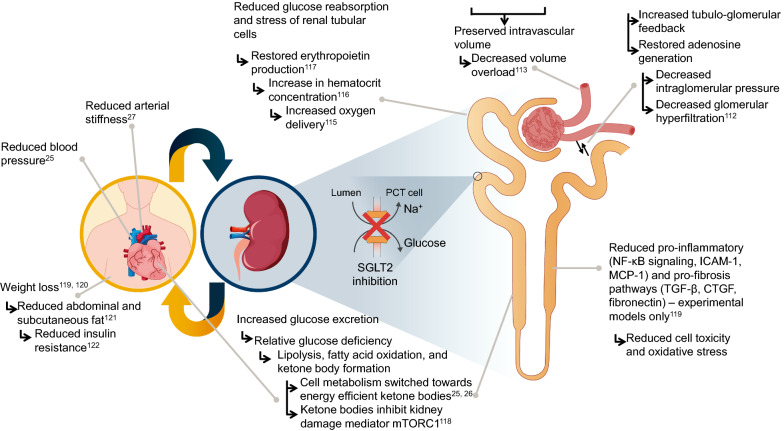
Proposed renal-protective pathways with SGLT2 inhibitors. *CTCF* connective tissue growth factor; *ICAM-1*, intercellular adhesion molecule 1; *NF-κB* nuclear factor kappa-light-chain-enhancer of activated B cells; *MCP-1* monocyte chemoattractant protein 1; *SGLT2* sodium–glucose cotransporter 2; *TGF-β* transforming growth factor beta

The inhibition of SGLT2 leads to tubuloglomerular feedback via enhanced natriuresis and delivery of sodium to the macula densa, thereby decreasing diabetic glomerular hyperfiltration due to hyperglycemia [112]. This is hypothesized to reduce the resulting chronic kidney damage (Fig. 3). In combination with the osmotic diuresis effect, natriuresis has also been postulated to preserve intravascular volume and decrease volume overload [113], although body fluid changes appear to be transitory [114]. Evidence from animal models suggests that SGLT2 inhibition reduces proinflammatory and profibrotic pathways, as well as tubular cell toxicity [115]. The significant increase in hematocrit concentration reported in patients receiving SGLT2 inhibitors [116] appears to be linked to increases in erythropoietin production [60, 117], which may also result in increased oxygen delivery to the kidney and reduction in renal hypoxia [115]. In addition, increased glucose excretion in the urine can lead to a state of relative glucose deficiency, triggering lipolysis in adipose tissue, fatty acid oxidation, and ketone body formation [25, 26]. Ketone bodies are a more energy-efficient fuel in renal tubular cells than glucose, and as a result, renal oxygen consumption is reduced in the presence of mild ketosis; hence, it has been suggested that the use of ketone bodies as energy substrates in patients receiving SGLT2 inhibitors may contribute to the renoprotective effects of these agents, with a similar mechanism as in the myocardium [25, 26]. Ketone bodies have also been found to inhibit the mechanistic target of rapamycin complex 1, a mediator of kidney damage in animal models [118]. In addition to hemodynamic effects, SGLT2 inhibitors also have beneficial metabolic effects that may contribute to renoprotection. Treatment with SGLT2 inhibitors leads to significant weight loss of up to approximately 5 kg [119, 120], two-thirds of which are accounted for by reductions in both abdominal and subcutaneous fat [121]. Indeed, after 6 months off treatment, weight loss has been found to be primarily due to loss of adipose tissue mass [114]. This fat mass loss can in turn lead to reductions in insulin resistance [122], metabolic risk [123], and renal risk [124].

The available SGLT2 inhibitors differ in their selectivity for SGLT2. While empagliflozin and dapagliflozin are highly selective for SGLT2, canagliflozin also inhibits SGLT1 to a small extent [125, 126]. However, whether SGLT selectivity may contribute to different renal benefits is currently unclear.

### Renal outcomes in studies with SGLT2 inhibitors versus studies with GLP-1 RAs

Both the GLP-1 RA and SGLT2 inhibitor classes have demonstrated significant glucose-lowering effects in people with T2D, with a low risk of hypoglycemia [127]. In addition, both have also shown significant benefits on renal outcomes. A reduction in the incidence of microvascular events (macroalbuminuria, doubling of serum creatinine, ESKD, or renal death) was reported with liraglutide, compared with placebo, in the LEADER CVOT (HR: 0.78; 95% CI 0.67, 0.92); these effects were similar irrespective of baseline eGFR or albuminuria [21, 128]. However, this reduction was primarily driven by lower rates of new-onset persistent macroalbuminuria (HR: 0.74; 95% CI 0.60, 0.91) [128]. A reduction in the risk of new or worsening nephropathy (macroalbuminuria, doubling of serum creatinine, eGFR < 45 mL/min/1.73 m^2^) with semaglutide compared with placebo was also reported in the SUSTAIN-6 CVOT (HR: 0.64; 95% CI 0.46, 0.88); again, this reduction was largely driven by reductions in macroalbuminuria (HR: 0.54; 95% CI 0.37, 0.77) [20]. The REWIND CVOT comparing dulaglutide with placebo found a reduction in the risk of the composite renal outcome (macroalbuminuria, eGFR decline of ≥ 30%, or chronic RRT; HR: 0.85; 95% CI 0.77, 0.93) following dulaglutide treatment, with a significant reduction in macroalbuminuria (HR: 0.77; 95% CI 0.68, 0.87) [129]. Similar to liraglutide, a *post-hoc* analysis demonstrated that the impact of dulaglutide on the renal composite outcome did not appear to be influenced by baseline eGFR or albuminuria status, although dulaglutide appeared to only prevent the onset of new macroalbuminuria in patients with eGFR < 60 mL/min/1.73 m^2^ (HR: 0.70; 95% CI 0.59, 0.81; *P* for interaction = 0.046) [130]. In addition, a sensitivity analysis found a 44% reduction in the number of patients with a sustained eGFR decline of ≥ 50% (HR: 0.56; 95% CI 0.41, 0.76) in the dulaglutide arm compared with placebo [130]. Somewhat in contrast to these findings, AWARD-7, a randomized, open-label trial of people with T2D and moderate-to-severe CKD (stage 3–4) reported that dulaglutide reduced the decline in eGFR and albuminuria compared with insulin glargine, particularly in patients with macroalbuminuria at baseline [131].

In a meta-analysis that combined three SGLT2 inhibitor CVOTs with five trials of GLP-1 RAs involving 42,920 patients, both GLP-1 RAs and SGLT2 inhibitors significantly reduced the risk of progression of kidney disease including macroalbuminuria compared with placebo, with hazard ratios of 0.82 (95% CI 0.75, 0.89) and 0.62 (95% CI 0.58, 0.67), respectively. The effect of SGLT2 inhibitors was significantly greater than that of GLP-1 RAs (*P* = 0.01 for heterogeneity). Furthermore, the positive effect seen with GLP-1 RAs seemed to be largely confined to progression to macroalbuminuria, while SGLT2 inhibitors reduced the risk of worsening eGFR, ESKD, or death from kidney disease (HR: 0.55; 95% CI 0.48, 0.64); GLP-1 RAs had no significant effect on this particular composite (HR: 0.92; 95% CI 0.80, 1.06) [132].

## Safety of SGLT2 inhibitors in T2D and renal impairment

The CVOTs with SGLT2 inhibitors and meta-analyses of these trials have shown that SGLT2 inhibitors have a reassuring safety profile in people with T2D, and the real-world experience is consistent with this [133]. The principal adverse effects in CVOTs include genital and urinary tract infections, which are to be expected owing to the glycosuric effects resulting from SGLT2 inhibition; however, meta-analyses found increased rates of genital but not urinary tract infections; the absolute numbers were low and infections were usually easily managed [44, 45]. There is also some evidence to suggest that combination treatment with SGLT2 inhibitors and DPP-4 inhibitors or GLP- RAs might reduce the incidence of such events [134, 135]. SGLT2 inhibitors produce a transient, dose-dependent reduction in eGFR [59, 112], which is reversible on stopping treatment [136]; additionally, real-world data show that SGLT2 inhibitors are not associated with an increased risk of AKI [137, 138]. CVOT analyses actually show that there may be fewer AKI events in patients on SGLT2 inhibitor therapy compared with placebo [42]. Despite previous signals for increased diabetic ketoacidosis in people with T2D treated with SGLT2 inhibitors, the overall risk appears to be low [139–142]. Although the CANVAS program found that canagliflozin was associated with significant increases in the risks of amputations or fractures [22], no such findings have been reported in trials with other SGLT2 inhibitors [23, 24, 32], the CREDENCE renal outcomes trial with canagliflozin [33], or a recent cohort study [143]. Meta-analyses also confirmed no overall increase in amputation or fracture risk with SGLT2 inhibitors, but did highlight significant heterogeneity between trials [44, 45, 144, 145]. Similarly, the use of SGLT2 inhibitors does not seem to be associated with increased rates of Fournier’s gangrene (necrotizing fasciitis of the perineum); although six cases were reported in DECLARE-TIMI 58, five of these occurred in the placebo group [23, 133].

In a meta-analysis of 27 studies, including approximately 7300 patients, treatment with SGLT2 inhibitors in people with T2D and CKD was not associated with increased risks of adverse renal events (HR: 1.04; 95% CI 0.68, 1.61), AKI (HR: 0.69; 95% CI 0.45, 1.06), or hyperkalemia (HR: 0.63; 95% CI 0.48, 0.83), compared with controls [45]. Rates of hyperkalemia were also reduced in the CREDENCE trial of people with T2D and CKD (HR: 0.80; 95% CI 0.65, 1.00) [33].

### Are renal outcomes with SGLT2 inhibition consistent in elderly patients?

Theoretically, the effects of SGLT2 inhibitors might differ in elderly and younger patients, owing to age-related decline in CV and renal function. The adverse event profiles of SGLT2 inhibitors may be slightly more pronounced in elderly patients. An analysis of six randomized controlled trials with canagliflozin found that some adverse events occurred more frequently in elderly patients (> 75 years), with higher rates of osmotic diuresis-induced effects and urinary or genital mycotic infections [146]. In STELLA-ELDER, a Japanese post-marketing study of ipragliflozin in elderly patients (age ≥ 65 years; 31% of patients were > 75 years of age), 16.9% of patients experienced adverse events, most commonly skin and subcutaneous tissue disorders, and renal and urinary disorders [147]. In a subgroup analysis of the STELLA-LONG TERM study, the overall incidence of adverse events was similar in patients aged < 65 years and in older patients (10.8% versus 10.4%, respectively), but the incidence of renal adverse events was significantly higher in older patients (0.5% versus 1.0%) [148].

Hypovolemia and dehydration may expose frail individuals to orthostatic hypotension or postural dizziness; it is therefore important to note that SGLT2 inhibitors do not induce a significant increase in sympathetic nerve activity in response to osmotic diuresis [149]. Hypotension and dehydration could potentially decrease renal filtration, leading to acute kidney failure that may be exacerbated by recurrent and/or unresolved kidney injury [150]. However, the current data are reassuring in this respect: after an initial decrease due to transient changes in renal hemodynamics, GFR stabilizes at values similar to baseline levels [30, 98], even in patients with moderate eGFR impairment at baseline and in elderly individuals [85, 151–153]. Furthermore, the available evidence suggests that SGLT2 inhibitors are not associated with dehydration in elderly patients [154, 155].

## Conclusions

The renoprotective effects of SGLT2 inhibitors do not seem to be influenced by baseline CV risk, including heart failure status. They are generally seen over a wide range of eGFR and albuminuria categories; however, it is difficult to elucidate whether these effects are greater in those with preserved or reduced renal function. In the EMPA-REG OUTCOME trial, the impact of empagliflozin treatment on the rates of acute renal failure and AKI was greatest in patients with baseline eGFR < 60 mL/min/1.73 m^2^ [24]. In the CANVAS program, canagliflozin was shown to reduce renal outcomes consistently across different levels of baseline albuminuria, but with the largest absolute benefits in those with macroalbuminuria [53]. DECLARE-TIMI 58 demonstrated that dapagliflozin attenuated the increase in UACR over time to the greatest extent in patients with the highest degrees of albuminuria [36]. In the CREDENCE trial, there was greater risk reduction in the renal-specific outcome with canagliflozin compared with placebo, in patients with UACR > 1000 mg/g and in patients with an eGFR < 60 mL/min/1.73 m^2^ [33]. In a meta-analyses of these CV and renal outcome trials, the renoprotective effects of SGLT2 inhibitors were seen across all levels of baseline eGFR, although the largest benefits were apparent in patients with preserved renal function at baseline [42].

Studies such as CREDENCE, DERIVE, and DAPA-CKD offer a greater insight into the renoprotective effects of SGLT2 inhibitors in patients with moderate-to-severe CKD than the CVOTs that preceded them. In addition, real-world studies such as CVD-REAL 3 report similar results to those observed in CVOTs of SGLT2 inhibitor therapies in patient cohorts with an ample range of baseline renal function. Together, the outcomes of these trials demonstrate how the benefits of SGLT2 inhibitors, used in combination with RAS inhibitors, span the renal risk continuum, from patients with mild/moderate CKD (as seen in the EMPA-REG OUTCOME, CANVAS program, and DECLARE-TIMI 58 trials) to those with moderate/severe CKD (as observed in CREDENCE and DAPA-CKD). Results across studies vary, but overall, suggest that different clinically utilized therapeutic doses have similar efficacy in terms of renal outcomes. Further insights into the potential benefits of SGLT2 inhibitors in patients at different stages on the renal and CV risk continuum are likely to come from the EMPA-KIDNEY (NCT03594110) trial in patients with diabetic as well as nondiabetic CKD.

## Data Availability

Data sharing is not applicable to this article as no datasets were generated or analyzed during the current study.

## Abbreviations

AKI: Acute kidney injury
BMI: Body mass index
CI: Confidence interval
CKD: Chronic kidney disease
CV: Cardiovascular
CVD: Cardiovascular disease
CVOT: Cardiovascular outcomes trials
DPP-4: Dipeptidyl peptidase-4
eGFR: Estimated glomerular filtration rate
ESKD: End-stage kidney disease
GFR: Glomerular filtration rate
GLP-1 RA: Glucagon-like peptide-1 receptor agonist
HR: Hazard ratio
NNT: Number needed to treat
OR: Odds ratio
RAS: Renin–angiotensin system
RRT: Renal replacement therapy
SD: Standard deviation
SE: Standard error
SGLT2: Sodium–glucose cotransporter 2
T2D: Type 2 diabetes
UACR: Urinary albumin:creatinine ratio
UK: United Kingdom
UKPDS: UK Prospective Diabetes Study
WMD: Weighed mean difference
